# Computed tomography and barium swallow findings of esophageal squamous cell carcinoma with suspected bronchoesophageal fistula in a patient with chronic graft-versus-host disease: A case report

**DOI:** 10.1016/j.radcr.2026.08.076

**Published:** 2026-09-12

**Authors:** Amirhossein Soltani, Amir Hossein Imani, Mohammad Hossein Imani

**Affiliations:** a Medical Imaging Research Center, Shiraz University of Medical Sciences, Shiraz, Iran; b Department of Radiology, Shiraz University of Medical Sciences, Shiraz, Iran; c Student Research Committee, School of Medicine, Shiraz University of Medical Sciences, Shiraz, Iran

**Keywords:** Esophageal squamous cell carcinoma, Chronic graft-versus-host disease, Hematopoietic stem cell transplantation, Bronchoesophageal fistula, Contrast esophagography, Secondary malignancy

## Abstract

Secondary esophageal squamous cell carcinoma is an uncommon but recognized late malignancy after allogeneic hematopoietic stem cell transplantation, particularly in patients with chronic graft-versus-host disease. We report a 33-year-old man with T-cell lymphoma treated approximately 8 years earlier with chemotherapy, radiotherapy, and allogeneic hematopoietic stem cell transplantation, complicated by chronic cutaneous and ocular graft-versus-host disease. He developed 6 months of progressive dysphagia and weight loss; upper endoscopy with biopsy confirmed esophageal squamous cell carcinoma, and he completed 6 cycles of preoperative chemotherapy. When severe oral intolerance prompted admission, barium swallow demonstrated an irregular short-segment mid-thoracic esophageal stricture at the carina with mild proximal dilatation and approximately 2.5 cm of linear contrast extravasation toward the left bronchial tree, raising suspicion for a bronchoesophageal fistula. Chest computed tomography showed an approximately 70-mm elongated mid-esophageal mass with proximal fluid-filled dilatation. Contemporaneous neck and abdominopelvic computed tomography showed no definite distant metastasis. No bronchoscopy or repeat endoscopy confirming the fistula was documented; the fistula therefore remained suspected. Esophagectomy was planned; nutritional consultation recommended parenteral support, and central venous access was established. The patient subsequently deteriorated and died after cardiopulmonary arrest before any documented esophagectomy. This case emphasizes the complementary roles of contrast esophagography and computed tomography and the importance of distinguishing malignant obstruction and fistulization from benign graft-versus-host disease-related esophageal strictures in post-transplant patients with new or progressive dysphagia.

## Introduction

Long-term survivors of allogeneic hematopoietic stem cell transplantation have an increased risk of subsequent solid malignancies. In a large Japanese cohort, the standardized incidence ratio for esophageal cancer after allogeneic transplantation was 8.5, and extensive chronic graft-versus-host disease was independently associated with esophageal cancer (relative risk, 5.3) [1]. An international cohort similarly showed that the incidence of solid cancers rises with time after transplantation and identified chronic graft-versus-host disease as an important determinant of subsequent squamous cell carcinoma risk [2]. Prolonged immune-mediated tissue injury and immunosuppressive treatment are considered contributors to this excess risk [3,4].

Esophageal disease in this population poses a diagnostic challenge because chronic graft-versus-host disease can itself cause dysphagia and benign structural abnormalities, including webs, ringlike narrowings, and tapering strictures [5]. Conversely, secondary esophageal squamous cell carcinoma may develop years after transplantation and can present with progressive dysphagia [6,7]. We report the complementary barium swallow and computed tomography findings of biopsy-proven esophageal squamous cell carcinoma with a suspected bronchoesophageal fistula in a young adult with chronic graft-versus-host disease after allogeneic transplantation.

## Case presentation

A 33-year-old man with a history of T-cell lymphoma diagnosed approximately 8 years earlier presented with severe progressive dysphagia. His previous treatment included chemotherapy, radiotherapy, and allogeneic bone marrow transplantation. The available medical record documented chronic graft-versus-host disease with cutaneous and ocular involvement: scars from prior graft-versus-host disease-related skin ulceration were present on the back and neck, and his ophthalmic medications included topical cyclosporine. No prior esophageal involvement by graft-versus-host disease was documented.

Approximately 6 months before the current admission, he developed progressively worsening dysphagia to both solids and liquids, accompanied by substantial weight loss, weakness, and malaise. Upper endoscopy identified an esophageal lesion, and histopathologic examination of an endoscopic biopsy confirmed squamous cell carcinoma. The record indicates that he subsequently received 6 cycles of chemotherapy in preparation for surgery; the last cycle was administered approximately 2 weeks before the acute presentation.

During the 10 days before admission, he became unable to tolerate either solid food or liquids. On admission, he appeared cachectic and pale. His heart rate was 121 beats/min and blood pressure was 90/72 mm Hg. Laboratory tests showed leukocytosis (white blood cell count, 18,000/µL), C-reactive protein of 57 mg/L, erythrocyte sedimentation rate of 74 mm/h, and alkaline phosphatase of 373 U/L. (Table 1)

**Table 1:** Clinical timeline.

Time	Clinical event	Key finding/management
∼ 8 y before current admission	T-cell lymphoma treated with chemotherapy, radiotherapy, and allogeneic bone marrow transplantation.	Later medical records document chronic cutaneous and ocular graft-versus-host disease.
∼ 6 mo before admission	Progressive dysphagia to solids and liquids with weight loss.	Upper endoscopy and biopsy confirmed esophageal squamous cell carcinoma.
Before acute admission	Six cycles of chemotherapy administered with surgery planned.	Last cycle approximately 2 wks before acute presentation.
December 22, 2025	Barium swallow for worsening dysphagia.	Irregular mid-thoracic stricture and ∼ 2.5 cm linear contrast extravasation toward the left bronchial tree; suspected bronchoesophageal fistula.
Late December 2025	Chest, neck, and abdominopelvic computed tomography; admission for severe oral intolerance.	∼ 70-mm mid-esophageal mass with proximal dilatation; no definite distant metastasis; no direct fistulous tract documented on available computed tomography images.
During terminal admission	Preoperative cardiology and nutritional evaluation.	Esophagectomy documented as planned; parenteral nutritional support recommended and central venous access established.
January 2026	Clinical deterioration and cardiopulmonary arrest.	Resuscitation unsuccessful; death occurred before any documented esophagectomy.

**Fig. 1:**
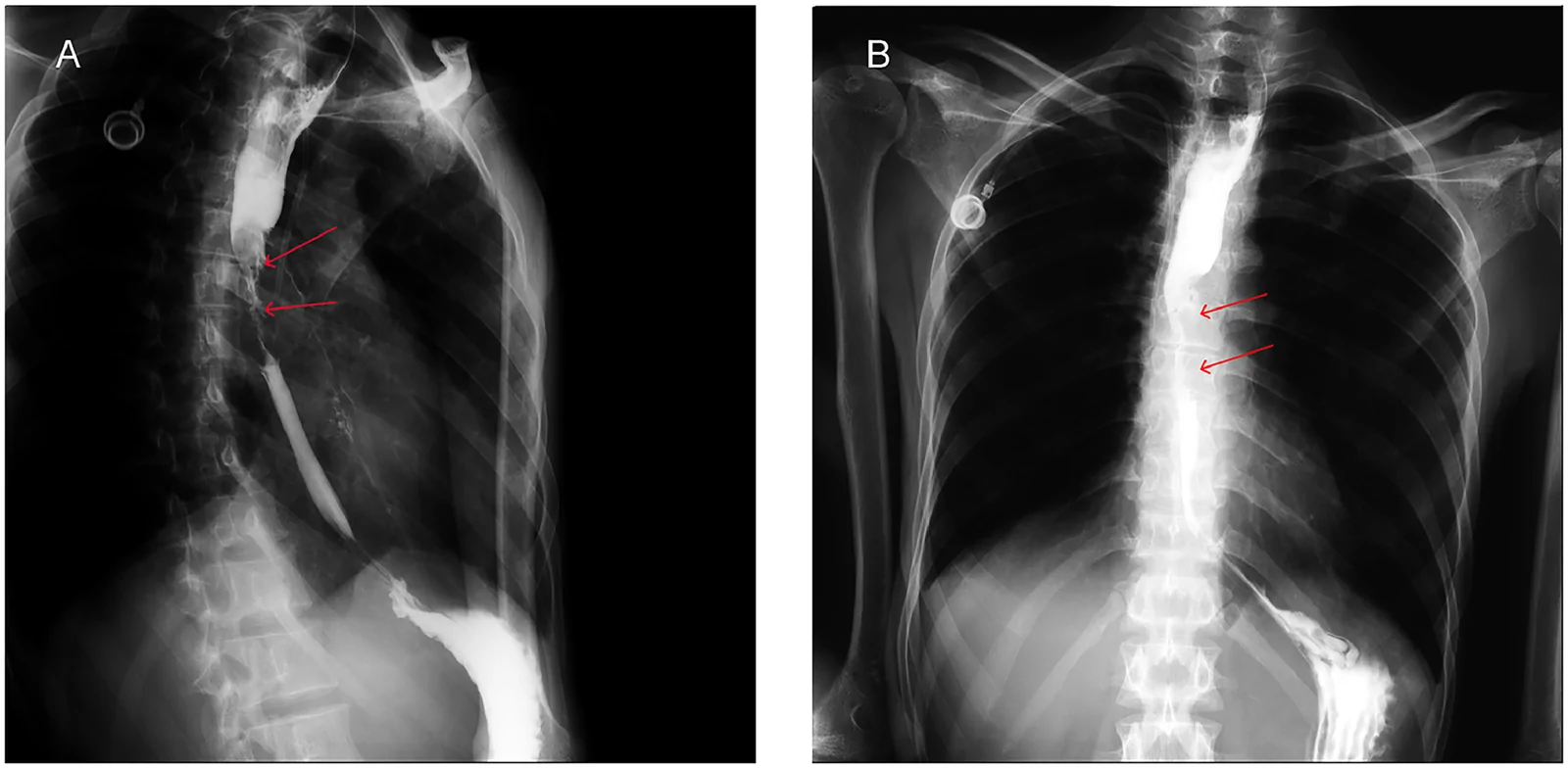
Barium swallow examination. Left image: left anterior oblique view demonstrates the irregular high-grade mid-thoracic esophageal stricture and the adjacent subtle linear contrast tracking described in the contemporaneous fluoroscopic report (red arrows). Right image: frontal view demonstrates irregular short-segment narrowing of the mid-thoracic esophagus at approximately the level of the carina, with proximal dilatation (red arrows). The fluoroscopic report described approximately 2.5 cm of contrast extravasation toward the left bronchial tree and concluded that a bronchoesophageal fistula should be considered; however, no bronchoscopic or repeat endoscopic confirmation was documented.

Barium swallow performed on December 22, 2025, demonstrated marked mucosal irregularity and a short-segment stricture in the mid-thoracic esophagus at the level of the carina, with mild upstream dilatation. The contemporaneous radiology report described approximately 2.5 cm of linear contrast extravasation toward the left bronchial tree with bronchial opacity and concluded that a bronchoesophageal fistula should be considered (Fig. 1). Because no subsequent bronchoscopy or repeat endoscopy confirming a fistulous opening was documented in the available record, the finding is described throughout this report as a suspected bronchoesophageal fistula rather than a confirmed fistula.

**Fig. 2:**
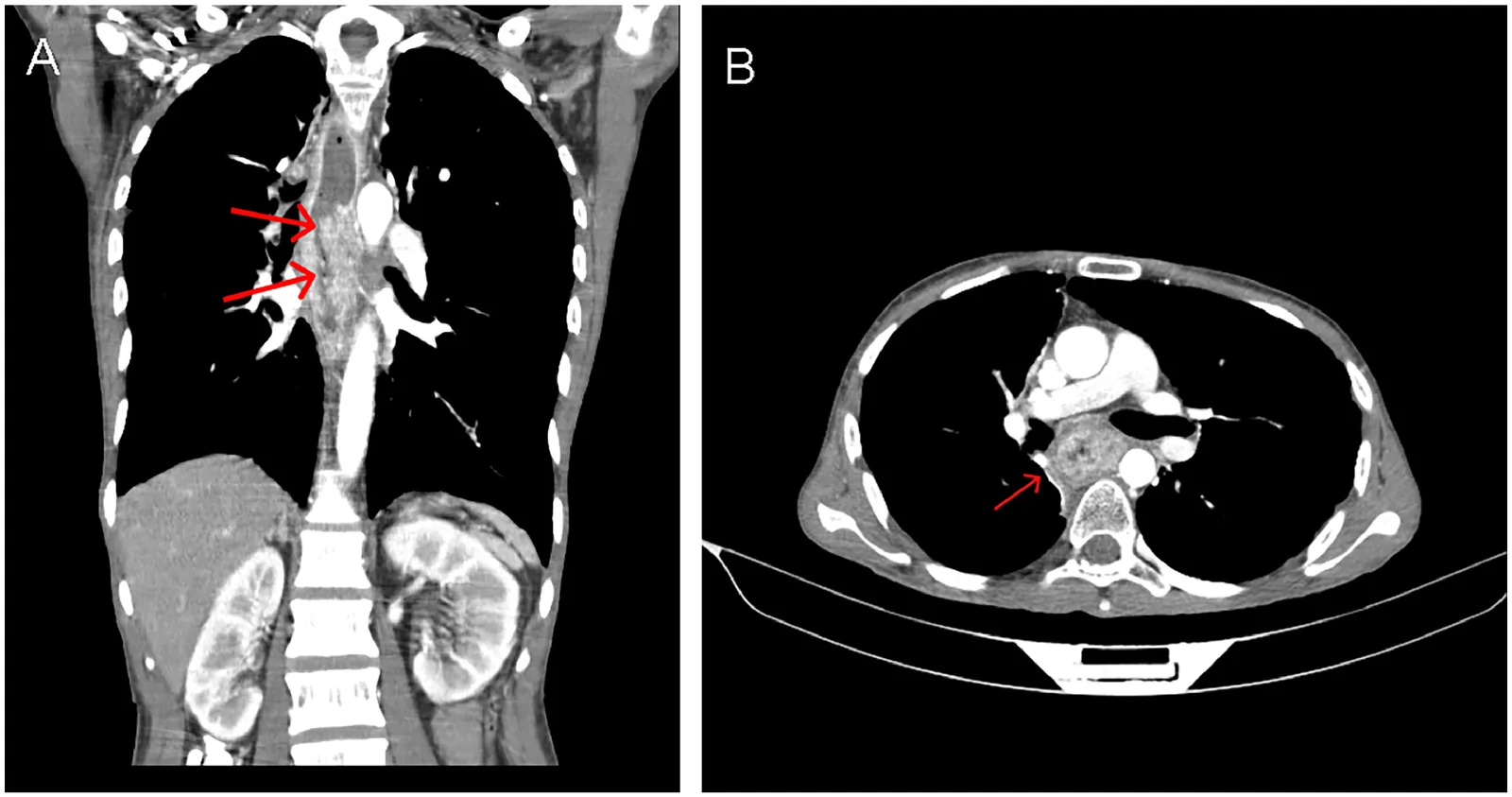
Contrast-enhanced chest computed tomography. Left image: coronal reconstruction demonstrates elongated irregular mid-esophageal soft-tissue thickening/mass in the subcarinal region (red arrows). Right image: axial image through the same level demonstrates the esophageal soft-tissue central airways (red arrow). A definite direct fistulous tract is not shown on the available computed tomography images.

Contrast-enhanced computed tomography of the chest demonstrated hyperinflated lungs, bilateral irregular subpleural reticulation suggestive of underlying interstitial lung disease, and right paramediastinal cicatricial bronchiectatic change that the radiology report considered possibly post-radiation. The upper third of the esophagus was fluid-filled and dilated. A heterogeneous elongated mass measuring approximately 70 mm in craniocaudal length and 20 mm in total thickness involved the mid-third of the esophagus (Fig. 2). No pleural effusion or mediastinal or hilar lymphadenopathy was reported. The available computed tomography images did not demonstrate a definite direct fistulous tract, and the official report did not assign a T category or describe definite invasion of an adjacent organ. Therefore, no T category or definite airway invasion is assigned in this report.

Contemporaneous computed tomography of the neck showed no abnormal cervical mass or lymphadenopathy. Abdominopelvic computed tomography showed no definite hepatic, adrenal, or other distant metastasis and no para-aortic lymphadenopathy. The bilateral subpleural reticulation described on the chest computed tomography is nonspecific and is therefore not attributed definitively to pulmonary graft-versus-host disease.

A perioperative cardiology consultation documented a planned esophagectomy. However, no operative report documenting esophagectomy was present in the available record. Because of severe weight loss and inadequate oral intake, a nutrition consultation recommended parenteral nutritional support, and central venous access was established. The patient subsequently deteriorated during the same hospitalization and sustained cardiopulmonary arrest. Resuscitation was unsuccessful, and he died before any documented esophageal resection. The available record does not establish that the suspected fistula specifically caused the terminal deterioration or that it directly changed the planned operation, so no such causal claim is made.

## Discussion

This case illustrates a clinically important late complication in a young allogeneic transplant survivor: biopsy-proven esophageal squamous cell carcinoma arising approximately 8 years after transplantation in the setting of chronic graft-versus-host disease. The timing is consistent with published experience. Nomura et al. identified esophageal squamous cell carcinoma in 17 of 1534 allogeneic transplant recipients (1.1%), with a mean interval of 82.3 months from transplantation to detection [6]. In a more recent institutional series of secondary esophageal cancer after hematopoietic stem cell transplantation, the median interval to diagnosis was 11 years [7]. Population-level data are also compelling: Atsuta et al. reported an 8.5-fold standardized incidence of esophageal cancer after allogeneic transplantation and a 5.3-fold relative risk associated with extensive chronic graft-versus-host disease [1].

The pathogenesis is likely multifactorial. Chronic graft-versus-host disease produces sustained immune-mediated epithelial injury and fibrosis, while prolonged immunosuppressive therapy can impair immune surveillance; these mechanisms have been linked to post-transplant squamous cell cancers [[2], [3], [4]]. Treatment-related exposures may also contribute. Nomura et al. found total body irradiation to be more frequent among patients who later developed esophageal squamous cell carcinoma [6]. The present patient had a history of radiotherapy for T-cell lymphoma, but the available record does not specify the radiation field or dose. It is therefore appropriate to regard prior radiation as a possible cofactor rather than to attribute this esophageal malignancy directly to mediastinal irradiation.

A key diagnostic issue is that dysphagia in chronic graft-versus-host disease does not necessarily indicate malignancy. Radiographic descriptions of esophageal graft-versus-host disease include webs, ringlike narrowings, and tapering strictures in the mid and upper esophagus [5]. In this patient, prior esophageal graft-versus-host disease was not documented. More importantly, the progressive dysphagia and weight loss were accompanied by an irregular focal stricture and an endoscopically biopsied squamous cell carcinoma, establishing a malignant cause of the obstruction. The benign graft-versus-host disease-related stricture nevertheless remains an important differential diagnosis for radiologists evaluating similar post-transplant patients.

The 2 imaging modalities provided complementary information. Contrast esophagography remains a primary radiographic examination for suspected esophago-airway fistula, while computed tomography can depict the esophageal mass, surrounding mediastinum, pulmonary complications, and direct or indirect signs of fistulization [8]. In the present case, fluoroscopy described linear contrast leakage toward the left bronchial tree, whereas the available computed tomography images did not show a definite direct tract. Published reviews emphasize that flexible esophagoscopy and bronchoscopy provide confirmation and anatomic assessment when a fistula is suspected [9]. Because such confirmation was not documented here, the terminology was deliberately limited to “suspected bronchoesophageal fistula.”

A malignant esophago-airway fistula has major implications for nutrition, aspiration risk, and treatment planning, and covered self-expanding stents are frequently used when definitive surgical management is unsuitable [9]. Selected transplant survivors with secondary esophageal cancer can undergo esophagectomy, including after neoadjuvant therapy [7,10]. In this patient, the record documents a planned esophagectomy and a preoperative cardiology assessment, followed by intensive nutritional support and central venous access. However, no esophagectomy or fistula-specific stenting procedure was documented before the patient's terminal deterioration. The available data therefore support stating that imaging identified a potentially management-relevant complication, but they do not support claiming that imaging definitively changed treatment.

## Teaching point

In patients with previous allogeneic hematopoietic stem cell transplantation and chronic graft-versus-host disease who develop new or progressive dysphagia, radiologists should consider both benign graft-versus-host disease-related stricture and secondary esophageal malignancy. An irregular focal stricture or mass warrants prompt tissue diagnosis, and contrast esophagography should be scrutinized for leakage into the airway. Computed tomography should define the mass and evaluate for fistulous and extraesophageal complications. When no endoscopic, bronchoscopic, or surgical confirmation is available, a suspected bronchoesophageal fistula should not be reported as confirmed.

## Declaration of generative AI and AI-assisted technologies in the writing process

During the preparation of this work the authors used ChatGPT (OpenAI) to assist with language editing.

## Patient consent

Written informed consent for publication of this case report and the accompanying images was obtained from the patient. The authors confirm that the patient was informed about the publication of clinical information and imaging findings, and that all reasonable efforts have been made to protect the patient's identity.
